# Supplementary figures and images for: Semi-supervised associative classification using ant colony optimization algorithm
Source: PeerJ Comput Sci. 2021 Sep 10;7:e676. doi: 10.7717/peerj-cs.676 (PMC8444075; doi:10.7717/peerj-cs.676)

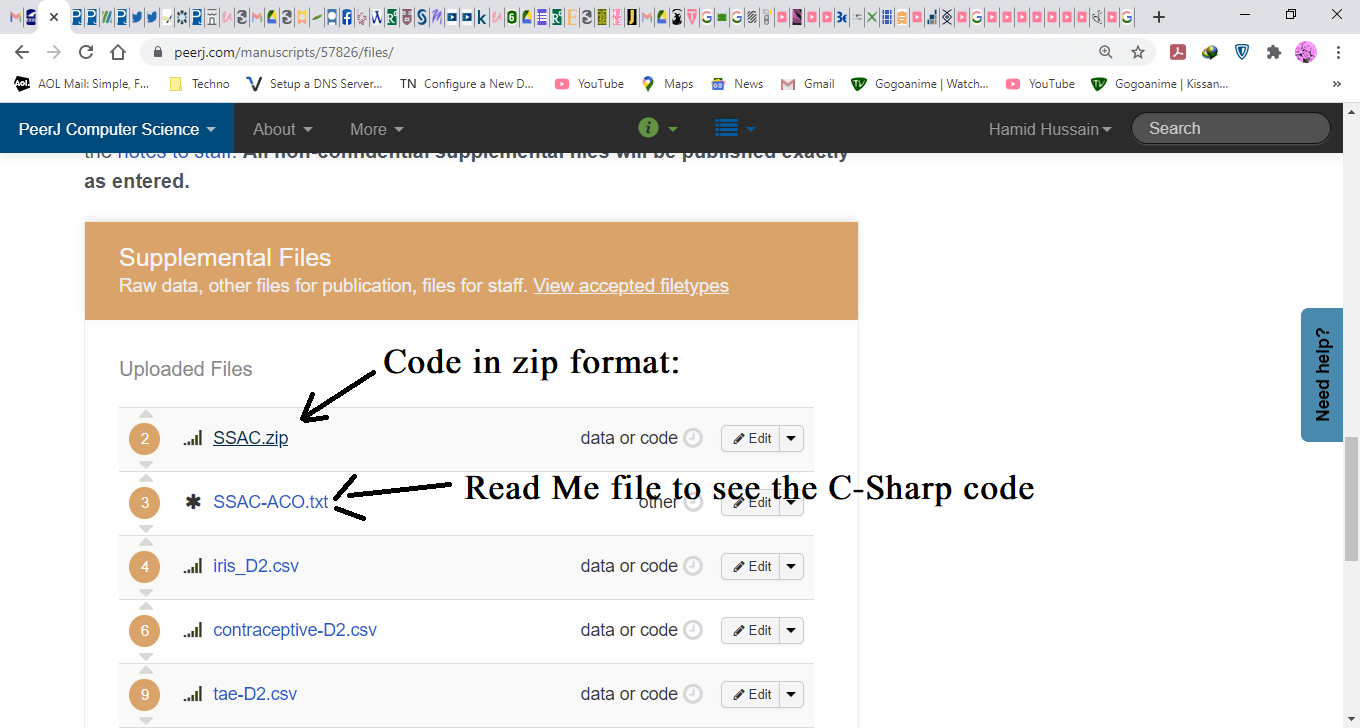

Supplement: Supplemental Information 3 [file peerj-cs-07-676-s003.png]

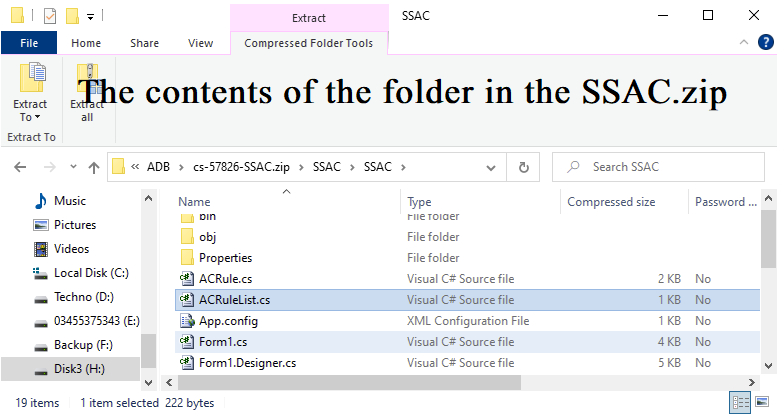

Supplement: Supplemental Information 4 [file peerj-cs-07-676-s004.png]
